# Exogenous amdoparvoviruses (*Parvoviridae*) in arvicoline voles: the molecular evolution and ecology of a novel host-viral association

**DOI:** 10.1371/journal.ppat.1013896

**Published:** 2026-01-22

**Authors:** Joseph A. Jackson, Mike Begon, Janette E. Bradley, Ida M. Friberg, Sarah Hyde, Klara M. Wanelik, Steve Paterson

**Affiliations:** 1 School of Science, Engineering and Environment, University of Salford, Salford, United Kingdom; 2 Institute of Infection, Veterinary and Ecological Sciences, University of Liverpool, Liverpool, United Kingdom; 3 School of Life Sciences, University of Nottingham, Nottingham, United Kingdom; 4 School of Biosciences, University of Surrey, Guildford, United Kingdom; University of Utah, UNITED STATES OF AMERICA

## Abstract

Amdoparvoviruses are best known as agents of disease in carnivorans, but here we provide the first in-depth molecular evolutionary and ecological information for an amdoparvovirus in wild rodents (field voles, *Microtus agrestis*). We applied an RNA-sequencing approach in lung tissue that yielded high diagnostic sensitivity and multiple full or near-full coding sequences for the new virus (field vole amdoparvovirus, FVAV) in individual voles. FVAV is most similar to amdoparvoviruses in European foxes and wildcats. We present evidence that FVAV is an exogenous, endemic, high-prevalence infection with a short-term history of horizontal transmission and recombination within voles and arising from an ancestral background of dynamic host usage and inter-lineage recombination. FVAV molecular structures involved in host exploitation share a highly conserved functional and evolutionary pattern with those in other amdoparvoviruses. The more variable regions within these structures evolve principally by apparently neutral processes and FVAV within-population mutation distribution mirrors that across the *Amdoparvovirus* phylogeny. Nonetheless, we did find some evidence of adaptive selection in the most variable regions and we also found convergent host-specific features in the modelled capsid protein of divergent arvicoline-associated lineages that might tend to restrict host range and support that FVAV is a vole-specialist. Increasing FVAV expression was associated with pulmonary inflammation and suppressed splenic T-cell activation, consistent with a potential to drive disease processes as in other amdoparvoviruses. Importantly, our approach highlights the *de novo* sequence assembly of viral RNA products from shotgun sequencing of rRNA-depleted RNA from tropic tissues in individual hosts as a sensitive and robust means of detecting and characterising not only RNA viruses but also DNA viruses.

## Author summary

Amdoparvoviruses are small DNA viruses usually associated with significant disease in carnivorans. Here we confirm the endemicity of a novel, exogenous amdoparvovirus in a rodent. We provide detailed evolutionary and ecological information for this new virus (field vole amdoparvovirus, FVAV), which occurs at high prevalence in the lungs of arvicoline rodents (field voles) in Northern England. We quantified infection and characterised the nucleic acid sequences coding for viral proteins via RNA-sequencing of lung tissue from individual hosts. Our work highlights this approach, targeting viral RNA products, as a good prospect for the discovery and characterisation of novel respiratory viruses of similar type, which hitherto may have been under-sampled. From our sequencing data we were able to reconstruct multiple full viral coding sequences, allowing molecular evolutionary analyses in relation to other members of the *Amdoparvovirus* genus. This revealed that FVAV is most closely related to amdoparvoviruses infecting wildcats and foxes in Northern Spain. Evidence of frequent recombination (which requires within-host contacts) amongst amdoparvoviral lineages, and their varied host usage, may reflect evolutionary host-switching. On the other hand, FVAV displays convergences of capsid structure with distantly related endogenized amdoparvoviruses that also occur in arvicoline (vole-like) hosts, suggesting it is an arvicoline specialist.

## Introduction

In this study we provide the first detailed phylogenomic and molecular ecological information on an exogenous amdoparvovirus persisting in a wild rodent population (field vole amdoparvovirus, FVAV, infecting the field vole, *Microtus agrestis*, in Northumberland, Northern England). Hitherto, amdoparvoviruses have been best known as highly transmissible agents of disease in wild and captive carnivorans [1–3] with some, thus far limited, level of zoonotic potential [4–6]. They are part of the wider *Parvoviridae* family [7] that includes the agent of the canine parvovirus type 2 panzootic that emerged in domestic dogs in the 1970s and spread worldwide within 1–2 years [8]. Several distinctive *Amdoparvovirus* lineages have so far been described within carnivorans [9] and additional lineages have been detected in bats [10–12]. Whilst amdoparvoviruses are thought to occur naturally in rodents [9,13], until now this possibility has been based solely on their discovery as endogenized elements [13] or on their detection [14,15] in metagenomic studies with little biological context. The existence of endogenized elements in rodent genomes [13], in particular, is consistent with the long-term recirculation of amdoparvoviruses in these hosts [9], which we are able to throw new light on in the present study.

*Amdoparvovirus*, as in other *Parvoviridae* genera, contains small DNA viruses with a c. 4.5 kb linear, single-stranded genome that codes for capsid (VP) and non-structural (NS) proteins. Different versions of the VP and NS proteins are expressed via alternative splicing of a single pre-mRNA molecule [16]. The earliest known *Amdoparvovirus* species was discovered as the agent of Aleutian Mink Disease [17,18] (Aleutian mink disease virus, AMDV; or *Amdoparvovirus carnivoran1* under International Committee on Taxonomy of Viruses, ICTV, nomenclature [19]), which causes serious and highly contagious outbreaks in farmed mink. AMDV has been studied in some detail, including characterisation of viral messenger RNAs (mRNA) [20], providing a valuable exemplar for interpreting the biology of other members of the genus.

AMDV causes a spectrum of acute and chronic disease states including respiratory disease and an immunologically-based progressive wasting syndrome [2]. Other amdoparvoviruses in carnivorans, such as racoon dog and fox amdoparvovirus (*Amdoparvovirus carnivoran3)* [21], skunk amdoparvovirus (*Amdoparvovirus carnivoran4*) [22] and red panda amdoparvovirus (*Amdoparvovirus carnivoran5*) [23] are associated with partially similar patterns of disease, indicating a generalised potential for adverse effects on the host within the wider *Amdoparvovirus* genus. AMDV viral particles are highly resistant in the environment [24] and transmission is remarkably flexible. Spread of infection may be vertical (transplacental [25]) or horizontal via multiple routes, including aerosol droplet-respiratory transmission and oral transmission through exposure to infected biological fluids or faeces, either via direct contact with infected animals or fomites. Long distance aerosol transmission [26,27] and arthropod transmission [28] are considered to be additional possibilities. Indeed, one of the bat-infecting *Amdoparvovirus* lineages, Sabeidhel virus (SBEHV1; *Amdoparvovirus chiropteran1*) has been detected in bat flies [10] which suggests the possibility of vectoring by biting arthropods.

Acute respiratory disease in AMDV results from the infection of pneumocytes [29] that may be invaded via interactions with sialic acid. On the other hand, chronic disease phenotypes appear to stem from the invasion of macrophages, which is possible via an antibody-dependent, Fcγ II-mediated mechanism [30,31] (a form of antibody-dependent enhancement, ADE). The non-specificity of this mechanism, whereby antibody binds to the capsid which is then internalised via interaction between the antibody and the Fc receptor, could predispose the virus to cross-species transmission. Indeed, AMDV infects a wide range of hosts, in the wild, in captivity, or following experimental infection, including mustelids, procyonids, mephitids, viverrids, felids, canids and murids [2,32]. It has also been known to infect human mink farmers in a few cases [5], causing chronic, ultimately lethal disease, although sustained sequences of human-to-human transmission have never been reported.

The present study is primarily based on the analysis of pulmonary transcriptomes in 38 *M. agrestis* individuals sampled over 13 months (2016–2017) at 5 sites in the Kielder Forest in Northumberland, North-East England. Although amdoparvoviruses are DNA viruses, nonetheless their mRNA products proved to be well represented in these transcriptomes and could be assembled into putative transcripts like some of the common mRNA species reported for AMDV. Moreover, as the samples were sourced from a wider study of infection and immunity in the field voles at Kielder [33,34], we are able to draw on a background of detailed information to make additional inferences about the evolution and biology of the new virus.

We present evidence below that FVAV is a high prevalence, endemic infection in field voles with a proximal history of horizontal transmission and intraspecific recombination and arising from an ancestral background of dynamic host usage and interspecific recombination. The new virus is most closely related to amdoparvoviruses found in European red foxes and wildcats and further research is required to establish whether FVAV is a vole specialist or is additionally able to exploit the carnivoran predators of the vole. More generally, our focus on the detection and sequence assembly of viral RNA products in shotgun-sequenced rRNA-depleted RNA from the tissues of individual hosts may represent a sensitive and underappreciated mode of viral discovery in wildlife [35]. This simultaneously detects DNA- and RNA-viruses and provides useful information about the interaction with the host when population samples are analysed.

## Materials and methods

### Ethics statement

As previously described [34], all animal work for this study was performed with approval from the University of Liverpool Animal Welfare Committee and under a UK Home Office licence (PPL 70/8210 held at the University of Liverpool) in accordance with the Animals (Scientific Procedures) Act 1986.

#### Field site and sampling.

Field voles (n = 38) were collected by live-trapping in the Kielder Forest, Northumberland, UK as previously described [34,36–39] between July 2016 and August 2017. Samples were derived from five established trapping sites: BLB (55.24262, -2.620250), CHE (55.21921 -2.543800), GRD (55.18404 -2.584000), HAM (55.23379 -2.585970) and SCP (55.26515 -2.545460) enclosing a c. 2500 hectare area on the western side of Kielder Water. After capture, individual animals were isolated for 1–2 days in filter-top plastic cages with bedding and *ad libitum* access to food and water. Thereafter the animals were killed by terminal anaesthesia followed by exsanguination and dissected in a Class II safety cabinet employing aseptic technique. Samples of cardiac blood and lung tissue were collected from each animal and conserved in RNA stabilisation solution (RNAlater; ThermoFisher, UK) following the manufacturer’s recommendations and transferred to a -80°C freezer for long-term storage. Further samples of cardiac blood and liver were flash frozen in liquid nitrogen and stored at -80°C for biochemical assays. Spleens were collected for each animal and processed for splenocyte culture as described below. All animals were weighed at the beginning of the period of captivity and weighed again and measured (snout-vent length) immediately prior to dissection. Sex, maturity (adult/juvenile) and weights for the liver and spleen were recorded during the dissection. In addition, a packed cell volume was determined for a small subsample of cardiac blood, as previously described [39].

#### Lung RNA sequencing.

Total RNA was extracted from conserved field vole lung samples and used to construct DNA-free rRNA-depleted (non-mRNA-selected) multiplexed paired-end libraries (2 × 150 base pairs) via the Azenta Life Sciences Standard RNA-Seq service. The libraries (1 library per host) were sequenced on an Illumina NovaSeq 6000 machine to yield 24–57 million (mean = 31 million) read pairs per sample. De-multiplexed FASTQ files received from the sequencing service were pre-processed employing *fastp* [40].

#### Viral metatranscriptomic detection and quantification.

To detect viruses, the RNA sequencing reads for each animal were first mapped to the Ensembl *Microtus ochrogaster* genome (MicOch1.0) with *BBMap* [41] (*Microtus ochrogaster* being a close relative of *M. agrestis*). Unmapped reads were saved and assembled employing *SPAdes* [42]. The resulting contigs were searched against the NCBI [43] RefSeq viral genomes database via discontiguous megablast [44] at a 40% identity cut-off, with only the strongest hit per contig considered. Substantial alignments (> 400 bp) were further investigated via *blastn* searches against the NCBI nt database. Contigs robustly attributable to the *Amdoparvovirus* genus were identified in many animals. Subsequently reads from all animals were re-mapped against a set of assembled contigs with *Salmon* [45], using MicOch1.0 as a “decoy-away” index, to provide a measure of amdoparvoviral read abundance. Further investigation of individual reads in the lowest abundance samples confirmed that genuine amdoparvoviral viral reads were present.

#### Viral mRNA sequence analysis.

Amdoparvoviral contigs recovered here were found to show conservation and synteny with reported mRNA species for AMDV, allowing the coding genomic and protein sequences of the VP and NS proteins to be robustly inferred from the mRNA data alone. Vole amdoparvoviral sequences were aligned with those for other amdoparvoviruses according to translated codons employing *ClustalW* [46]. FVAV sequences were also aligned against each other to identify polymorphic sites. Aligned sequences were clustered in *MEGA12* [47] via the Maximum Likelihood method employing an optimal substitution model determined to have the lowest Bayesian Information Criterion (BIC) [48] amongst many candidate standard models. The initial tree for heuristic searches was chosen via the default settings in MEGA12 and all analyses excluded positions with gaps or missing values. For both VP and NS, we analysed *Amdoparvovirus*-wide alignments based on the available full or near-full coding regions and also alignments based on smaller regions in order to include additional viruses for which only genomic fragments were available. The inclusion of sequences was predicated upon their availability in GenBank at the time of analysis. We included one sequence per nominal lineage (the ICTV-named sequence in the case of ICTV-recognised species). This was except in the case of FVAV and its closest relatives where in some analyses we included all available sequences. Predicted domains and functional sites within FVAV VP and NS were inspected with CD-search [49,50] and PROSITE [51] and manually. Analyses of recombination and selection were carried out on the *Datamonkey 2.0* server [52,53] employing alignments of FVAV VP and NS full coding sequences (for variant 3) with those full or near-full coding sequences included in the phylogenetic analyses described above (but excluding endogenized viruses and the highly divergent BtR1-PV/FJ2012). To detect recombination, the Genetic Algorithm for Recombination Detection (GARD) [54] was applied specifying 3 rate classes and beta-gamma site-to-site variation [55]. Alignments partitioned according to the inferred recombination breakpoints were then analysed with the bootstrapped fixed-effects likelihood method (FEL) [52,56] to test for natural selection at individual alignment sites. This analysis specified synonymous rate variation and branch-specific rates for double and triple substitutions and tested at the FVAV branch with a *P* = 0.1 cut-off. We additionally applied GARD analysis to alignments of FVAV VP and NS sequences to assess the possibility of recombination within the FVAV lineage. Evolutionary rates across the *Amdoparvovirus*-wide alignments for full or near-full sequences (see above) were estimated position-by-position by maximum likelihood using the rate function in *Mega 12* [47]. Fisher exact tests [57] were used to test the independence of the distribution of signatures of selection amongst domains and other sequence features using the *R* function *fisher.test*. Differences in the by-position evolutionary rate between domains and other features were tested with Kruskal-Wallis [58] tests using the *R* function *kruskal.test*. Clustering of polymorphisms or signatures of selection along the amino acid sequence of proteins was tested against the expectation of a uniform distribution with Kolmogorov-Smirnov tests [59] employing the *R* function *ks.test*.

#### Molecular modelling of VP1.

FVAV VP1 was modelled with SWISS-MODEL [60,61], employing a previously published [62] electron microscopy structure (SMTL ID: 8ep2.1) for AMDV VP1 as a template. VP1 structures in other amdoparvoviruses were studied comparatively by also modelling these on the same AMDV template, thus standardising the comparison. The resulting models were aligned to and superimposed upon the FVAV model using the structure comparison and visualisation functions in SWISS-MODEL. QMEANDisCo Global [63] model quality values were 0.74-0.79 for the non-AMDV models. Regions of structural conservation or non-conservation were determined by per-residue consistency values between compared structures and a consensus structure. These are defined as the average Cα atom based lDDR (Local Distance Difference Test) [64] score which reflects differences in pairwise interatomic distances up to 0.1 nm distances. To contrast the consistency of different viruses in comparisons with FVAV we applied a Generalised Additive Model for Location, Scale and Shape (GAMLSS) [65] with a beta distribution inflated at 1 (BEINF1) [66,67] employing the *gamlss* library in *R* version 4.3.2. The BEINF1 distribution was suitable because the molecular structure at the majority of aligned residues was highly conserved, with a consistency of 1. Intercepts only were fitted for the *σ* (scale) and *ν* (probability of observation being 1) model parameters, with *ν* varying little because of shared regions of high conservation. For the μ (location) parameter (modelling differences in consistency values < 1) there was a fixed explanatory term for virus identity and a random term for sequence position. Differences of consistency between viruses were interpreted via parameter *t*-*t*ests setting mole vole endogenous amdoparvovirus as the reference level.

#### Host pulmonary transcriptomic analysis.

Using the lung RNA sequencing results described above, we analysed differential expression of host genes in relation to FVAV mRNA expression. Analyses were carried out in *R* version 4.3.2. Pre-processed paired-end reads were mapped to the Ensembl *Microtus ochrogaster* (MicOch1.0) genome [68] (achieving 97–98% mapping) and enumerated (achieving 67–71% assignment to genomic features)) employing the *Rsubread* package [69]. The *M. ochrogaster* genome was used for mapping rather than the available *M. agrestis* genomes because these species are closely related and because the former is a longer-standing assembly with more bioinformatic resources available for downstream analyses. Following the exclusion of low expression genes (determined as those with <0.5 counts per million in >53% of samples), samples were normalised via a trimmed means of M-values (TMM) method [70] and subjected to differential expression (DE) analysis via a quasi-likelihood negative binomial generalized log-linear model in *edgeR* [71]. Only FVAV normalised expression was included as an explanatory variable, as site, season, host sex and host size were not found to be associated with FVAV expression (see below) and would have used up many degrees of freedom. The functions of gene products for genes that were significantly differentially expressed (*P* < 0.05) after false discovery rate (FDR) adjustment [72] were individually investigated via the *DAVID* gene functional classification tool [73], via *GeneCards* summaries [74] and through searches of journal literature in *PubMed* [43]. Gene Set Enrichment Analysis (GSEA) [75] was applied to the database of genes ranked by log fold change in the differential expression analysis using the *fgsea* library [76]. For this analysis we employed gene sets (see S1 Table) that might reflect broad immune responses (such as adaptive or innate immunity), antiviral responses (such as type 1 interferon responses) or antibody-dependent mechanisms considered to be important in AMDV (such as immune complex clearance). We also included some gene sets that might reflect health or disease in the lung (such as cell cycle, metabolism, pneumocyte development or pneumonia). In most cases the gene sets were based on *Mus musculus* genes associated with relevant Gene Ontology (GO) terms [77,78] (see S1 Table) but we also used the HP_PNEUMONIA gene set from the Human Molecular Signatures Database (MSigDB) [75,79] which is based on HP:0002090 in the Human Phenotype Ontology [80]. No other genes sets, apart from those listed in S1 Table, were used in the analysis. Vole gene identifiers for the GSEA analysis were obtained by filtering for the relevant GO terms or official gene names in Ensembl BioMart [68] for *Mus musculus* and returning orthologues for *Microtus ochrogaster*.

#### Host phenotypic markers.

Condition factors for total body weight, liver weight and spleen weight were calculated as the residuals of quadratic regressions of weight (at processing) upon body length. Packed cell volume of cardiac blood was measured as previously described [39]. We calculated proportional weight change during captivity from the weights recorded post-capture and at dissection. In addition, superoxide dismutase 1 (SOD1) antioxidant enzymatic activity was measured in blood (U/ml) as previously described [34]. Liver triglycerides (mg/g) were measured employing the Cayman Chemicals (Ann Arbor, Michigan) Triglyceride Colorimetric Assay kit (10010303) and liver glycogen (μg/g) with the BioVision (Milpitas, California) Glycogen Colorimetric Assay (K648-100), in both cases according to the manufacturer’s instructions.

#### Immunostimulatory assays with cultured splenocytes.

Splenocytes were isolated from whole spleens, cultured and harvested under conditions previously described [34,36]. In brief, from each animal, replicate cultures were established and exposed to different immunostimulants. These immunostimulants included: 1, combined anti-CD3 (Hamster Anti-Mouse CD3e, Clone 500A2 from BD Pharmingen, San Diego, California) and anti-CD28 (Hamster Anti-Mouse CD28, Clone 37.51 from Tombo Biosciences, Kobe, Japan) antibodies intended to specifically activate T-cells; 2, the mitogen PHA-L (Merck Life Science, Dorset) which also stimulates T-cells but is more non-specifically immunogenic; 3, the toll-like receptor 2 (TLR2) agonist HKLM (heat-killed *Listeria monocytogenes*) (tlrl-hklm, InvivoGen, San Diego, California); and 4, the toll-like receptor 7 (TLR7) agonist imiquimod (tlrl-imqs-1, InvivoGen). Unstimulated cultures (culture medium only) were also established to represent constitutive expression.

#### Expression of immune-associated genes in blood and cultured splenocytes.

Expression in panels of genes was measured by two-step quantitative reverse-transcription real-time PCR (QPCR) in whole blood and in cultured splenocytes. Methods for this and the selection of the target genes have been described in detail previously [34,39]. Briefly, a separate panel of genes, from varied pathways, was assayed in whole blood (28 genes) and splenocytes (21 genes). Most of the assayed genes have clear immune-associated functions and all could be considered of immune relevance as they would have been expressed in immunological cell populations in the current assays. Gene expression values analysed here are relative quantitation (RQ) values indexed to a calibrator sample and normalised to a pair of endogenous control genes by the ΔΔCt (2^-ΔΔCT^) method (see [34,39]).

#### Viral variants and host mitochondrial lineages.

Full coding sequences for the mitochondrial cytochrome c oxidase I (*mt-co1*) gene in individual hosts were assembled from the lung RNA sequencing reads above. For this, paired end reads were mapped to a *Microtus agrestis mt-co1* gene sequence (GenBank: MN487101.1) with *BBmap* and then saved and assembled employing *SPAdes*. For comparison to host *mt-co1* haplotypes, viral variants were initially characterised by Maximum Likelihood phylogenetic analysis (see above) of aligned regions of the more highly expressed R2 mRNA-like contigs. This was based on the 6 animals with full or near-full coding sequences. (We note that the resulting host-specific phylogeny was topologically consistent with that resulting from corresponding analyses of concatenated alignments for the R2 mRNA-like and R1’ mRNA-like contigs, or of R1’ mRNA-like contigs by themselves). Other animals with an R2 mRNA-like sequence fragment >400 bp were then assigned to one of the variants on the basis of further phylogenetic analysis. In each case, the sequence fragment was aligned with the full or near-full sequences and analysis carried out with complete deletion (all gaps and missing data removed). Variant identity was assigned where the fragment clearly clustered with one of the variants with bootstrap support (≥90%, or in one case 70%; and assignment was possible in all cases). The distribution of viral variants amongst mitochondrial haplotypes was visualised by a haplotype network plotted using the *pegas* library [81] in *R* version 4.3.2. To check whether multiple viral variants infected individual hosts we mapped reads from the 6 hosts with fully or near-fully assembled coding genomes and higher viral expression to an index of aligned R2 mRNA-like contigs for the different variants using *Salmon* (see above).

#### Linear modelling.

An initial linear statistical model was applied to the FVAV expression data to determine whether there were any associations with site, season, host size or host sex using the base *lm* function in *R* version 4.3.2. This model contained log_10_ (*x* + 1) transformed normalised FVAV expression as the response and fixed explanatory terms for host sex (factor 2 levels; M/F), site (factor 5 levels; BLB/CHE/HAM/GRD/SCP), season (factor 3 levels; summer-autumn 2016/spring 2017/summer 2017) and host length (continuous, mm). Full, nested and null models were compared via AICc [82].

#### Machine-learning analysis.

A machine learning analysis was applied to a combined set of 141 non-pulmonary host variables to evaluate their predictiveness for FVAV expression (these variables are listed in S2 Table). FVAV expression normalised to library size was set as the response in a Random Forest model [83,84] implemented using the *randomForest* function from the *randomForest* library in *R* version 4.3.2. The predictor variables included all host phenotypic markers and all gene expression variables described above. A small number of missing values amongst the predictor variables (3.5% of dataset) were imputed by proximity within the Random Forest analysis using the *rfImpute* function. We did not include host sex or body length in these analyses as they did not show any association with FVAV expression in the preliminary linear modelling analysis (see above). Significance of the overall model was evaluated via a permutation test (2000 permutations) in which the predictor variables were permuted by row. The most predictive variables were identified via ranking importance values (mean Increase in node purity). Given that a single category of variables (the gene expression variables in the CD3/CD28-stimulated group) was more predictive than others, we then asked what form was taken by the functional relationship of these variables with FVAV expression. To answer this, we implemented a linear mixed model (LMM) with Yeo-Johnson transformed gene expression values (for the top six predictive variables in the category) as the response, normalised FVAV expression (continuous) as a fixed explanatory term and gene, individual vole identity and QPCR assay plate (representing the assaying batch structure) as random terms. This employed the *lmer* function from in *lme4* package [85] (in *R* version 4.3.2) and tested the fixed term via Satterthwaite’s method in the *lmerTest* package [86].

#### Sequencing of the FVAV DNA genome.

To confirm the FVAV DNA genome we designed a set of 25 primers for FVAV variant 3 (S3 Table) that could be combined in different pairings to amplify fragments across the entire NS and VP coding regions and any intronic sequences within or between these. DNA was extracted from lung tissue (conserved in RNA stabilisation solution) from a host with relatively high mRNA expression of variant 3 using the DNeasy Blood & Tissue kit (Qiagen). For this, the manufacturer’s protocol for animal tissue was followed except that a bead mill was employed for mechanical homogenization at the lysis buffer step (5 mm stainless steel bead; TissueLyser II, Qiagen). Duplicated PCR amplifications with different primer pairings were carried out with MyTaq PCR mastermix (Meridian Bioscience) following the manufacturer’s suggested protocol and thermal cycling conditions, setting an annealing temperature of 53°C. PCR products from all duplicate reactions were pooled in approximately equimolar amounts and sequenced via Oxford Nanopore sequencing (Plasmidasaurus, Premium PCR Sequencing service) returning 10 × 10^3^ reads 91–5905 (mean = 577) base pairs in length. The reads were assembled from the FASTQ file provided by the sequencing service employing the *Canu* assembler [87].

#### Quantitative real-time PCR diagnostics targeting genomic DNA.

To compare the diagnostic value of the above transcriptomic observations to independent measurements we carried out real-time PCR amplifications targeting viral genomic DNA. Employing material from all of the same voles included in the transcriptomic study described above, DNA was independently extracted from newly excised 25 mg pieces of the original conserved lung tissue of each individual vole. DNA extraction was carried out as for the genomic sequencing described above. For real-time PCR we employed primers whose binding sites were conserved in all FVAV variants (L10 and R11; see S3 Table) and that generated a 67 base pair fragment of the VP gene. Assays were carried out with SYBR Green chemistry (PowerUp SYBR Green Master Mix, ThermoFisher) on a Quantstudio 6 real-time PCR system (ThermoFisher) in a 96-well plate format and employing the machine default cycling conditions for a comparative C_T_ experiment except that the annealing temperature was set at 53°C. In order to standardise for variation in extraction quality, we also, for each sample, ran amplifications for a host endogenous control gene (*actb*). Each sample was run in duplicate wells for each primer set. Each plate additionally included duplicate no-template controls for each primer set and a calibrator sample (an arbitrarily selected positive sample run on all plates). The results were expressed as relative quantitation (RQ) values, as if for a comparative C_T_ experiment, calculated using the Quantstudio 6 machine software, normalising to host *actb* and indexing to the calibrator sample via the ΔΔCt (2^-ΔΔCT^) method.

#### Evaluation of the possibility of contamination in the transcriptomic data.

Although all possible standard steps to prevent cross-sample nucleic acid contamination were employed, the possibility of some level of contamination cannot be precluded in a study of the current form, either in the case of transcriptomic or real-time PCR measurements. This might result from biological (infectious) contamination as animals are collected in the field and aggregated prior to processing, or it could be technical contamination during sample handling, nucleic acids extraction or sequencing library preparation. To quantify the amount of contamination in the transcriptomic sequencing we took advantage of the existence of unique FVAV sequences that it was possible to assemble from individual hosts with higher FVAV expression (see above and Results section below). Thus, all six hosts for which it was possible to assemble full or near-full coding regions showed unique coding sequences that could be assigned to one of 4 distinctive variants by phylogenetic analysis. Moreover in 11 further hosts where it was possible to assemble an FVAV fragment of > 400 base pairs, these could be assigned to one of the variants. As all of the assembled sequences contained unique variation in coding sequence, and mapping studies (see above) indicated that the 6 hosts with full or near-full assemblies were dominantly infected by virus of a single variant and contained negligible reads from other variants (<1% total of FVAV reads), we assumed that the highly expressed dominant unique sequences were true positives. We further reasoned that the small number of reads mapping to a non-dominant variant could result either from genuine infection or from contamination. Taking the most pessimistic view that all reads mapping to non-dominant variants in the 6 hosts with longer assemblies were due to contamination, we focussed on the two hosts containing dominant FVAV variants that were exclusively observed in those hosts (variants 1 and 2), as in these cases we could assume that the reads for the common variants (3 and 4) might be contaminants. (In contrast, for hosts infected with a common dominant variant, it would be unclear how many of the common variant reads would be true positives and how many would be false positive contaminants from other hosts infected with the common variant.) Taking the count of non-dominant reads in the hosts infected with variant 1 and variant 2 to represent contaminating reads, we derived a conservative cut-off below which we could much less confidently assign true infected status. This took the mean non-dominant variant read count (standardised to the mean library size) in the hosts of variant 1 and variant 2 and, assuming this would conform to a Poisson distribution (an appropriate distribution for random rare events), added two standard deviation units to give the cut-off.

#### Maps.

Maps were created in *R* version 4.3.2 employing the *ggmap* library [88] and an open data base map (GeoJSON file) published by the Department for Environment Food & Rural Affairs (DEFRA), UK, to represent the outline of Kielder Water.

## Results

### An exogenous amdoparvovirus in wild field voles, field vole amdoparvovirus (FVAV), is most closely related to amdoparvoviruses in European foxes and wild cats

**Fig 1:**
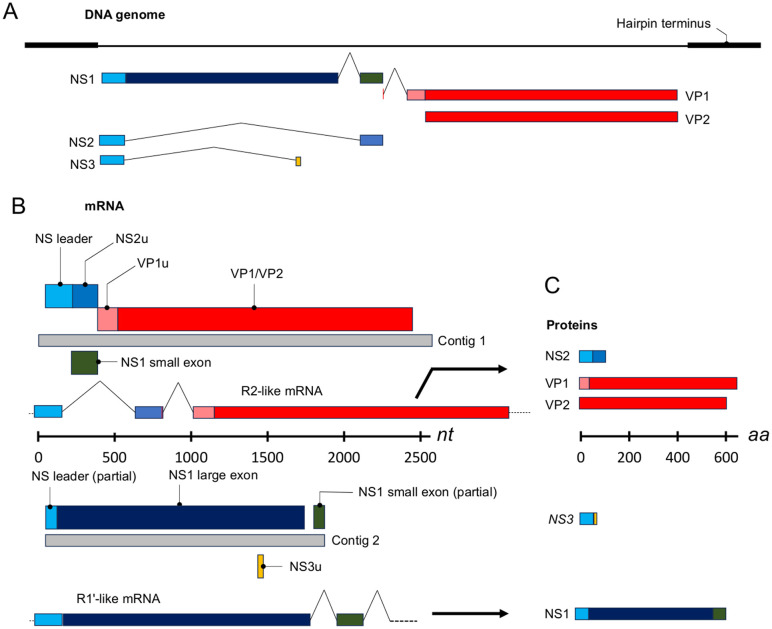
Field vole amdoparvovirus (FVAV) contigs derived via short read sequencing of total host lung RNA and *de novo* assembly. Showing the arrangement of coding sequences within the contigs and their relationship to putative mRNA species and to genomic and protein sequences. **A**. The arrangement of the FVAV DNA genome based on direct sequencing for the coding regions and central intronic regions and on the conserved pattern seen in other amodoparvoviruses for the termini [89]. Coding sequences are shown below coloured according to their contribution to NS and VP proteins. **B**. FVAV contigs approximated to the two most highly expressed Aleutian mink disease virus (AMDV) mRNA species, R2 and R1’ [20]. FVAV coding sequences (identified by sequence and splicing site homology), coloured according to the corresponding protein region, are shown in relation to the contigs (grey) and to the putative mRNA species (based on those documented in AMDV [20]). The putative mRNAs are also coloured by protein coding region. Likely open reading frames operational in the mRNA corresponding to the contig are shown above the contig, other overlapping reading frames are shown below. A “u” postscript indicates a region that is unique to a given protein. **C**. The proteins that would putatively be assembled from the coding sequences based on the conserved pattern in other studied amdoparvoviruses.

**Fig 2:**
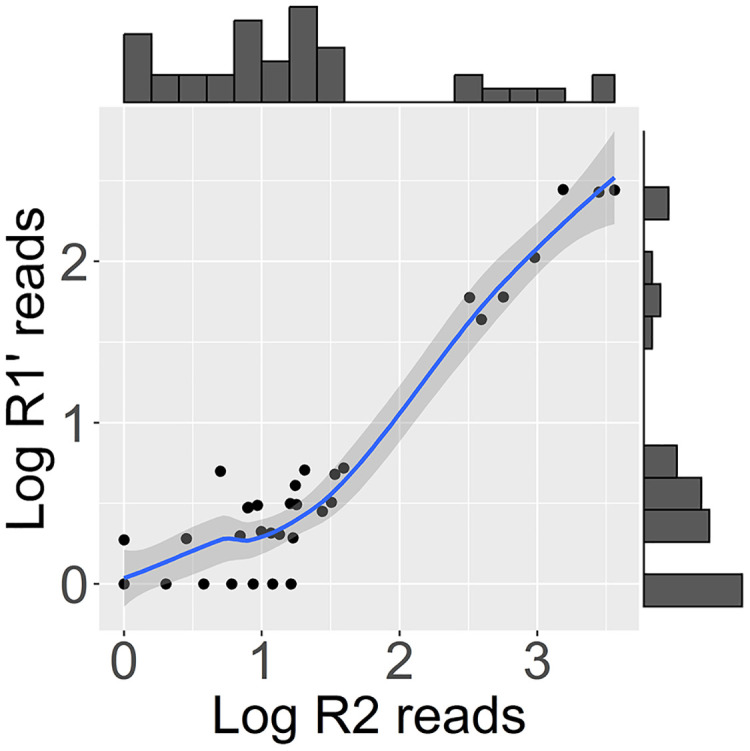
Frequency distributions and scatterplot for log-transformed (log_10_ *x* + 1) standardised reads mapping to the R2 mRNA-like and R1’ RNA-like field vole amdoparvovirus (FVAV) contigs. Based on shotgun sequenced pulmonary RNA from 38 field voles. A Loess smoother (blue) is shown with shaded 95% confidence interval. The read counts for the R2 and R1’ mRNA-like contigs were highly correlated (Spearman’s rho, *r*_*s*_ = 0.81) and the R2-like contig was typically expressed much more abundantly (up to an order of magnitude) than the R1’-like contig.

Illumina short-read sequencing of rRNA-depleted RNA from the lungs of 38 individual wild field voles in the Kielder Forest revealed the presence of a novel amdoparvovirus, field vole amdoparvovirus (FVAV). Six vole specimens yielded sufficient amdoparvoviral reads to cover the full coding sequence, or most of the coding sequence, for amdoparvovirus capsid (VP) and nonstructural (NS) proteins. *De novo* assembly in these cases produced two large contigs of c. 1800 (4–22 × coverage) and c. 2600 bp (15–195 × coverage) which respectively corresponded to the R1’ and R2 mRNA species previously described in AMDV (see Fig 1 and S4 Table) [20]. The expression of these contigs was highly correlated, but with the R2 mRNA-like contig being much more highly expressed, as in AMDV (Fig 2) [20]. In the case of the VP, intact open RNA reading frames (ORF) corresponding to a 685 aa VP1 and 641aa VP2 protein were recovered (Fig 1). In the case of NS, intact ORFs were recovered that corresponded to NS1 (654 aa), NS2 (114 aa) and NS3 (71 aa) proteins (Fig 1). For one host we sequenced the majority (4157 contiguous base pairs) of the FVAV DNA genome including all coding regions and interspersed intronic regions (GenBank: PX491703; S4 Table), confirming that these occur with the conserved synteny seen in previously known amdoparvoviruses (Fig 1).

**Fig 3:**
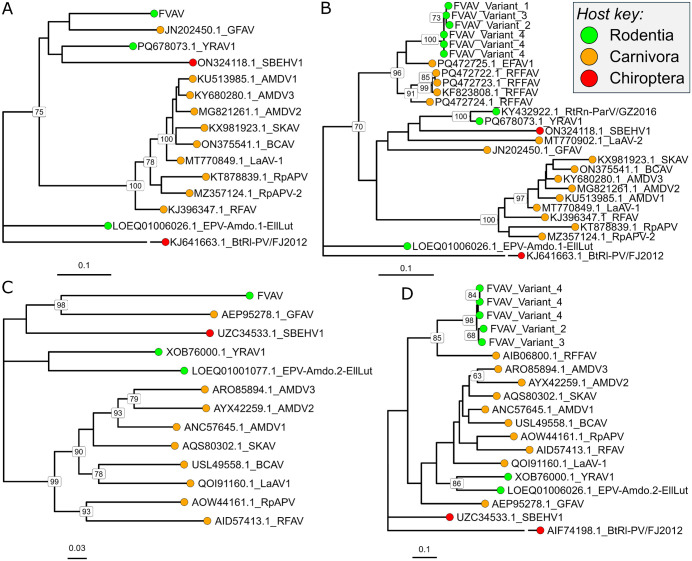
Relationships of field vole amdoparvovirus (FVAV) molecular sequences to those in other amdoparvoviruses based on Maximum Likelihood phylogenetic analyses. **A**. Nucleotide sequences coding for the VP1 protein in viruses for which full length or near full-length sequences were available. This analysis assumed a GTR + G substitution model and was based on 1905 positions. **B**. Nucleotide sequences coding for a smaller region of the VP protein allowing the inclusion of more viruses for which fragmentary genomic information was available. This analysis assumed a HKY + G substitution model and was based on 483 positions. **C**. Amino acid sequences for the NS protein in viruses for which full length or near full-length sequences were available. This analysis assumed an LG + G + I substitution model and was based on 562 positions. **D**. Amino acid sequences for a shorter region of the NS protein allowing the inclusion of more viruses for which fragmentary genomic information was available. This analysis assumed an LG + G substitution model and was based on 150 positions. **A-D**. AMDV1, Aleutian mink disease virus 1 (International Committee on Taxonomy of Viruses name: *Amdoparvovirus carnivoran1*); AMDV2, Aleutian mink disease virus 2 (*Amdoparvovirus carnivoran9*); AMDV3, Aleutian mink disease virus 3 (*Amdoparvovirus carnivoran10*); BCAV, British Columbia amdoparvovirus (*Amdoparvovirus carnivoran8*); BtR1-PV/FJ2012, bat parvovirus isolate; EFAV-1, European felid amdoparvovirus 1; EPV-Amdo.1-EllLut and EPV-Amdo.2-EllLut, endogenised mole vole amdoparvoviruses; GFAV, gray fox amdoparvovirus (*Amdoparvovirus carnivoran2*); LaAV-1, Labrador amdoparvovirus 1 (*Amdoparvovirus carnivoran6*); LaAV-2, Labrador amdoparvovirus 2; YRAV1, Yunnan rodent amdoparvovirus 1; RFAV, racoon dog and fox amdoparvovirus (*Amdoparvovirus carnivoran3*); RFFAV, red fox faecal amdoparvovirus; RpAV, red panda amdoparvovirus (*Amdoparvovirus carnivoran5*); RpAV-2, red panda amdoparvovirus 2 (*Amdoparvovirus carnivoran7*); RtRn-ParV/GZ2016, *Parvovirinae* sp. Isolate; SKAV, skunk amdoparvovirus (*Amdoparvovirus carnivoran4*); SBEHV1, Sabeidhel virus (*Amdoparvovirus chiropteran1*). Positions containing gaps or missing values were not considered. Trees with the maximum log likelihood are shown; scale bars indicate substitutions per site; bootstrap support [90] for nodes is indicated where this is above 60% (*n* = 500 replicates). GenBank sequence identifiers are included in the taxon labels as a prefix.

To establish the relationships of FVAV to other amdoparvoviruses we carried out Maximum Likelihood phylogenetic analyses of molecular sequences for both VP and NS (Fig 3). This was based on alignments for the available full or near-full sequences and also on alignments for smaller regions, in order to include viruses for which only sequence fragments were available. Analysis of the near-full nucleotide sequences for the more conserved VP region, and of amino acid sequences for the less conserved NS region, indicated that FVAV falls outside a well-supported cluster containing many carnivoran-infecting viruses, including AMDV and most other currently known lineages. Other deep-branching lineages falling outside this main carnivoran-infecting cluster have relatively poorly resolved interrelationships and include gray fox amdoparvovirus (GFAV; *Amdoparvovirus carnivoran2*) from New World canids, Sabeidhel virus (SBEHV1; *Amdoparvovirus chiropteran1*) from West African bats, Yunnan rodent amdoparvovirus 1 (YRAV1) [15] from Chinese murids and BtRl-PV/FJ2012 from Chinese bats. Endogenised amdoparvoviruses from the Transcaucasian mole vole also clustered outside the main carnivoran-infecting cluster and separately from FVAV. Corresponding (more inclusive) analyses of smaller regions clearly indicated a close relationship of FVAV to red fox faecal amdoparvovirus (RFFAV) and European felid amdoparvovirus 1 (EFAV-1) previously found in European red foxes and wildcats. These analyses were mostly consistent with the analyses of longer sequences above, but in the case of NS may have had limited resolution due to the shortness of the available alignment.

FVAV variants share 69–70% amino acid identity with the most complete reported NS1 fragment for RFFAV and 95–97% identity amongst themselves based on a maximally inclusive 153 position alignment. In comparison, there is 60–63% amino acid identity with GFAV (the next most identical lineage) across this same alignment. A full alignment (630 positions) between FVAV (variant 3) and GFAV indicates 65% identity and a longer alignment (561 positions) between FVAV variants indicates 95–96% identity amongst these. FVAV is very unlikely to be endogenous in nature as its sequences are absent in several full genomes reported for *Microtus agrestis* in the Kielder Forest (GenBank: GCA_902806775.1, GCA_902806765.2, GCA_001305995.1). Moreover, the intactness of the FVAV ORFs and many of the observations below are most consistent with an exogenous, active infection.

#### FVAV is a high-prevalence, endemic infection whose distribution is consistent with some element of horizontal transmission.

**Fig 4:**
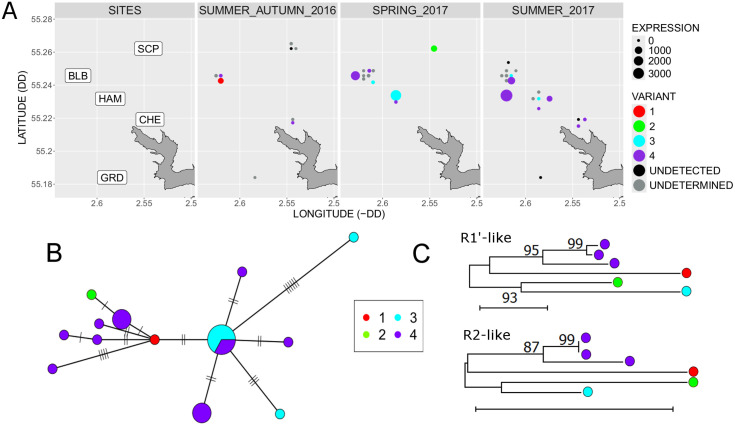
The distribution of field vole amdoparvovirus (FVAV) variants across spatiotemporal sampling points and host mtDNA matrilineages. **A.** The distribution of FVAV broken down by variant, time and locality within the Kielder Forest. Each point represents one host and is sized according to the abundance of viral mRNA reads (standardised to the mean library size of 31 million reads) and coloured according to the viral variant (see key); a small x-y jitter is applied to make points from the same site visible. DD: decimal degrees. Kielder water, excluding Bakethin Reservoir, is shown in the bottom right of each panel. The base map (https://environment.data.gov.uk/catchment-planning/WaterBody/GB30327698.geojson) contains public sector information licensed under the UK Open Government Licence v1.0.(see: https://www.nationalarchives.gov.uk/doc/open-government-licence/version/1/open-government-licence.htm). **B**. A host haplotype network for the cytochrome c oxidase subunit 1 (*mt-co1*) gene sequence with nodes (representing unique haplotypes) sized according to the number of hosts and coloured according to the frequency of occurrence of FVAV variants (see key). Nucleotide differences between haplotypes are indicated by ticks on the network edges and by edge length. **C**. Maximum Likelihood clustering of aligned nucleotide sequences for the R1’ mRNA-like contig and the R2 mRNA-like contig in FVAV. Analysis includes sequences from the six hosts with the highest viral expression and from which it was possible to assemble long contigs. Assuming an HKY + I substitution model and based on 1256 and 2329 positions respectively for R1’ and R2. Bootstrap node support [90] above 60% is indicated (based on 500 replicates); scale bar represents 0.01 nucleotide substitutions per site.

FVAV mRNA was detectably expressed in 89% (34/38) of voles. The distribution of expression was highly right-skewed (Fig 2) with a minority (18%, 7/38) of individuals presenting discontinuously higher FVAV read counts than others. Investigation of individual reads from low abundance samples via *blastn* searches and alignments confirmed that these were genuinely of amdoparvoviral origin. FVAV RNA expression was not associated with host length or sex, or with location or season (Fig 4). However, we note that sampling only took place during March-October and there were few (two) juvenile hosts in the sample and these were approaching maturity (a breakdown of *M. agrestis* sample characteristics is provided in S5 Table). Viral RNA expression was undetectable in several voles (11%, 4/38), including one of the two juveniles sampled. However, the continuous distribution of the lower read counts, extending smoothly to zero (Fig 2), prevented the confident assignment of uninfected status.

The full or near-full viral coding sequences extracted from six host specimens with high viral expression were all unique and could be assigned to 4 distinctive variants (Fig 4) based on phylogenetic analysis. A further 11 hosts yielded sufficient assembled sequence (also with unique mutations) to be assigned to one of these variants. The variants co-occurred across the study period and the most common variant (variant 4) was present throughout the study and at all of the more heavily sampled localities (Fig 4). Mapping of the variant identity onto a host mitochondrial DNA (*mt-co1*) haplotype network indicated that variant distribution broke across host mitochondrial matrilineages (Fig 4). This is consistent with at least some recent history of horizontal transmission, although vertical transmission is not ruled out. Coinfection with multiple FVAV variants in the hosts with higher viral expression (*n* = 6) was negligible or absent as read mapping analysis (employing *Salmon*) returned 99.3% assignment of reads to a single dominant variant in each host, even with the fractional assignment of multi-mapping reads. Furthermore, the DNA genome that was independently sequenced and assembled for one host was virtually identical in coding regions to transcriptomic assemblies for the same host (99.95% identity; i.e., two differences across all coding sequence) supporting the existence of a single dominant viral sequence within this host.

We then considered whether low FVAV read counts in mRNA could be an artefact of cross-contamination. We employed the existence of unique dominant FVAV sequences in some hosts as an internal control (i.e., a natural “spike-in”) to evaluate a “worst-case-scenario” possibility of cross-sample contamination in our transcriptomic samples (see Material and methods for full explanation). Even with a pessimistic cut-off (c. 0.5 reads per million, or about 15 reads in the average library), that assumed all reads from non-dominant sequences were contaminants, infection prevalence was still determined to be 47%. This figure corresponded approximately to the percentage of hosts (45%) in which it was possible to assemble a > 400 base pair mRNA fragment for FVAV. In contrast, our real-time PCR amplification of VP genomic DNA was potentially consistent with 100% infection (38/38 PCR positives). Moreover, the quantity of FVAV DNA in the samples normalised to host *actb* DNA had a moderate but significant correlation (Spearman’s rho, *r*_*s*_ = 0.48, *P* = 0.003, *n* = 36) with the VP transcriptomic reads (standardised for library size), even though these measurements were derived from different parts of the same lung and RNA copies would not necessarily be biologically expected to relate directly to DNA copies (i.e., perhaps depending on viral activity within a particular host).

### FVAV is associated with pulmonary inflammation

**Fig 5:**
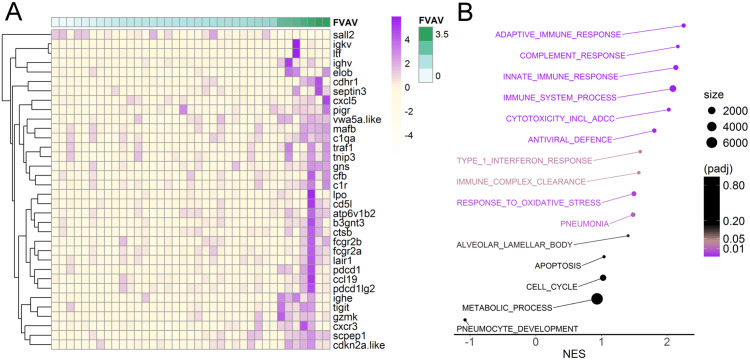
Field vole amdoparvovirus (FVAV) was associated with the elevated relative expression of immune-associated genes in the host pulmonary transcriptome. **A**. Heatmap showing the differential expression of host genes in individual voles with increasing FVAV expression. Host gene expression values (main left panel) are TMM normalised and scaled (zero mean, unit standard deviation) read counts for genes significantly differentially expressed after FDR-adjustment, almost all of which are immune-associated. FVAV expression (track at top of left panel, green) is in reads normalised to library size. **B**. Results from a GSEA analysis including 15 custom gene sets representing immunological and other organismal processes. Immune-associated gene sets were broadly upwardly differentially expressed, but this was not the case for other gene sets. Normalised Enrichment Scores (NES) (x-axis) for the gene sets analysed are represented by individual points that are coloured according to significance (padj) and sized according to gene set size.

Host transcriptomic reads (for 17369 annotated genes, after filtering those with low expression) were analysed to determine their association with the log-transformed relative abundance of FVAV reads. Thirty-four host genes were found to be differentially expressed after FDR correction, with almost all (97%, 33/34) of these being upwardly differentially expressed (Fig 5A and S6 Table). There was a remarkable preponderance (76%, 26/34) of genes with functions clearly relevant to immune responses (see S6 Table), all of which were upwardly differentially expressed. This immune-associated set included both the low affinity immunoglobulin gamma Fc receptor II genes (*fcger2a* and *fcger2b*). Other genes with products involved in antibody responses included immunoglobulin heavy and kappa chain variable regions, the immunoglobulin epsilon heavy chain, the polymeric immunoglobulin receptor (*pigr*) and antibody-fixing components of the classical complement system (*c1qa*, *c1r*). Further genes were associated with broadly based immune processes, including the alternative complement system (*cfb*), immune cell trafficking (*cxcl5*, *ccl19*, *cxcr3*, *b3gnt3*), regulation of lymphocyte activity (*mafb*, *cd5l*, *pdcd1*, *pdcd1lg2*, *tigit*, *lair*), cytotoxic lymphocyte effector responses (*gzmk*) and different aspects of innate immunity or inflammation (*ltf*, *lpo*, *traf1*, *gns*, *ctsb, tnip3*).

GSEA analysis confirmed a clear signature of upregulated general immune processes as FVAV expression increased (Fig 5B and S7 Table). This was clearest in broad genes sets (such as immune system process, innate immune response, adaptive immune response); but gene sets representing narrower immune responses, such as have been implicated in AMDV or that might be relevant for defence against viruses, were also significantly upregulated. The latter included gene sets representing cytotoxicity and antibody-dependent cytotoxicity (ADCC), Fc-receptor-mediated clearance of immune complexes via endocytosis, type 1 interferon responses and general antiviral defences. In contrast, gene sets that might reveal deep-seated disease progression (cell cycle, apoptosis, metabolic process, pneumocyte development) were unperturbed. Whilst gene sets for pneumonia and for response to oxidative stress were upregulated, leading-edge analysis indicated that this involved many immune-associated genes for pneumonia and oxidative stress is well known to be interconnected with inflammation [91].

### FVAV is associated with suppressed splenic T-cell responsiveness

**Fig 6:**
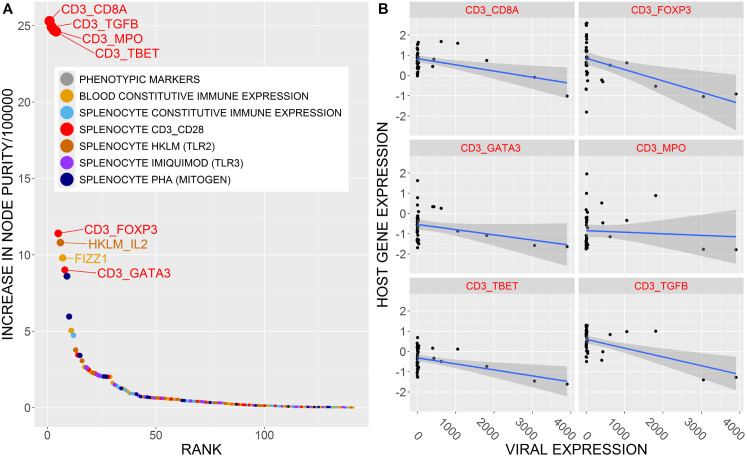
A machine learning algorithm (Random Forest) applied to 141 non-pulmonary host variables revealed a subtle negative association between field vole amdoparvovirus (FVAV) expression and the responsiveness of splenic T-cells. **A**. Ranked plot of variable importance in the Random Forest analysis with variable sets colour-coded by group (see key). High importance variables were mostly immune gene expression measurements in cultured splenocytes stimulated with anti-CD3 and anti-CD28 antibodies intended to activate T-cells. **B**. Plots of Yeo-Johnson transformed relative gene expression (RQ) in the highest importance variables in the CD3/CD28-stimulated splenocyte set against FVAV expression (x-axis, reads normalised to library size). Linear mixed model analysis (LMM) indicated a significant overall negative trend in CD3/CD28-stimulated splenocyte gene expression with respect to increasing FVAV expression.

Given the transcriptomic associations observed in the lung, we additionally asked whether FVAV expression was associated with a wider set of measurements relating to host physiological and systemic immune status. These measurements fell into several categories, including phenotypic markers such as condition indices and biochemical measures, constitutive expression for 28 immune-associated genes in the blood and expression for 21 immune-associated genes in splenocytes cultured under 5 separate conditions. These conditions were stimulation with anti-CD3 and anti-CD28 antibodies, or with mitogen, or with TLR2 agonist, or with TLR7 agonist, alongside an unstimulated control. Analysing FVAV expression as the response, we applied a Random Forest algorithm to this aggregated dataset (141 variables), finding a small but significant degree of predictiveness (7% variation explained; permutation *P* = 0.04). The highest variable importances were for CD3/CD28-stimulated splenocyte gene expression responses (Fig 6A and S2 Table) which would reflect T-cell activation. A secondary analysis to understand the form of the relationship between these predictive variables and FVAV expression found an overall negative effect of FVAV expression on splenocyte expression (LMM, β = -0.00032 ± 0.00006, *F*_1,220_ = 31.4, *P* < 0.0001) (see also Fig 6B).

### The FVAV capsid (VP) protein is highly conserved with fast-evolving externally-facing features

**Fig 7:**
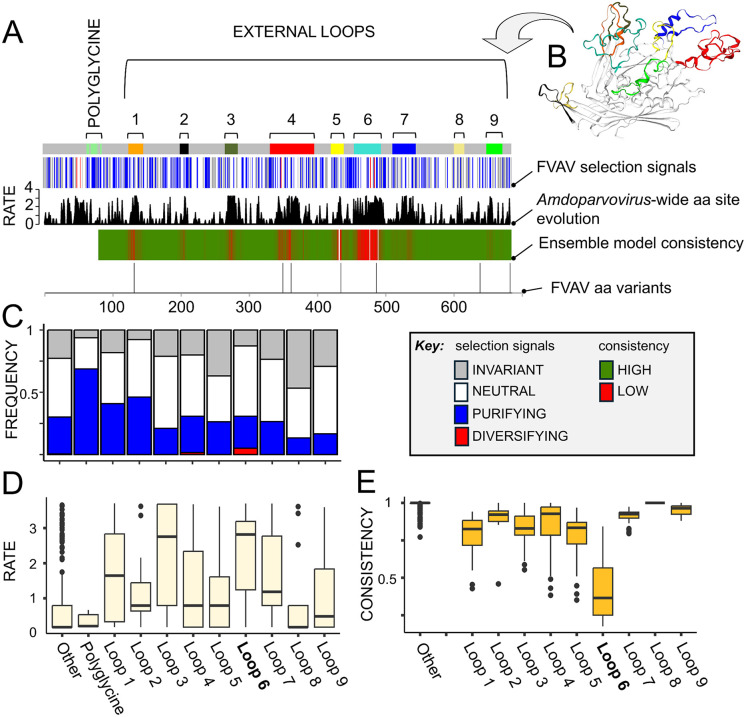
Structural variation in field vole amdoparvovirus (FVAV) VP1, including characterization of highly variable loop structures that are directed towards the exterior of the viral capsid oligomer. **A**. From the top, tracks show the distribution of: protein motifs and externally-directed loops; codon selection signatures; amino acid (aa) evolutionary rate derived from an *Amdoparvovirus*-wide alignment; consistency (see key) with an ensemble molecular model for Aleutian mink disease virus (AMDV1; International Committee on Taxonomy of Viruses name: *Amdoparvovirus carnivoran1*), Gray fox amdoparvovirus (GFAV; *Amdoparvovirus carnivoran2*) and FVAV (white indicates no data due to gaps); the distribution of amino acid polymorphisms in the Kielder Forest FVAV population. For the evolutionary rate track the y-axis indicates the relative rate in substitutions per site. **B**. A molecular model of FVAV VP1 colour-coded to show externally-directed loops. **C**. Stacked bar chart showing the relative frequency of different selection signatures (see key) across motifs, loops and other sequence regions. **D**. Box-and-whisker plots showing the distribution of evolutionary rate (derived from an *Amdoparvovirus*-wide alignment) across motifs, loops and other sequence regions. **E**. Box-and-whisker plots showing the distribution of FVAV consistency with an ensemble molecular model for AMDV, GFAV and FVAV across loops and other sequence regions. **A-E**. Loop 6 is characterised by particularly high evolutionary rate, low structural consistency and a high proportion of neutrally variable and diversified codons.

**Fig 8:**
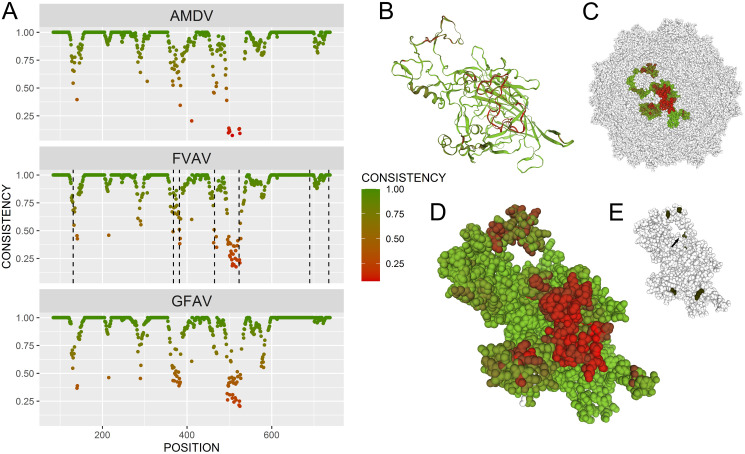
The field vole amdoparvovirus (FVAV) VP1 capsid protein contains regions of low structural conservation that correspond to loops facing externally in the capsid oligomer. Panels show the results of comparative molecular modelling of the VP1 protein in FVAV, Aleutian mink misease virus 1 (AMDV1; International Committee on Taxonomy of Viruses name: *Amdoparvovirus carnivoran1*) and Gray fox amdoparvovirus (GFAV; *Amdoparvovirus carnivoran2*). **A.** Residue-level consistency of species-specific models with an ensemble model averaging the models for all three species. Low structural conservation (low consistency) is concentrated in the same regions in all three species. The locations of amino acid polymorphisms found in the Kielder Forest FVAV population are shown by dashed lines. **B-E**. SWISS-MODEL molecular model structures for FVAV VP1, coloured according to residue consistency (see colour scale) (B-D) or location of polymorphisms (E) and in equivalent orientations. **B.** “Cartoon” representation (representing the protein backbone) of monomer. **C-E.** “Spacefill” representations (representing atoms as spheres, with sizes proportional to their van der Waals radii). **C.** VP1 molecule within the context of the capsid structure (note low consistency regions are externally directed). **D**. VP1 monomer. **E.** VP1 monomer showing positions of polymorphic residues (olive).

It was possible to robustly infer the structure of the VP1 and VP2 proteins from the R2 mRNA-like contig alone, on the basis of conserved splicing site residues, synteny and sequence homology with the genomes and mRNA species reported in other amdoparvoviruses (Figs 1 and 7). As in other amdoparvoviruses, FVAV VP1 lacks a phospholipase A2 domain and contains a polyglycine region (Fig 7). We carried out homology modelling of FVAV VP1 employing as a template the published model for AMDV VP1 [62], covering the C-terminal 80% of codon positions (GMQE = 0.77; QMEANDisCo Global = 0.77) (Fig 8). We also modelled GFAV VP1 in the same way and produced a consensus (ensemble) model for FVAV, GFAV and AMDV, assessing the consistency of the species-specific models with the ensemble model. This allowed us to evaluate regions subject to structural evolution (Fig 8). The three species-specific models revealed high consistency with the ensemble model across much of the molecule (Fig 8), although in some regions, whose location was highly conserved, consistency was dramatically lower (Fig 8). In the molecular models, these inconsistent regions code for externally-directed projecting loop structures in the polymeric viral capsid (Figs 7-8). Some of the low consistency loops were associated with elevated genus-wide evolutionary rates (Kruskal-Wallis test, *P* = 2.7 × 10^-8^), as has been reported in AMDV, in which the variable regions have been termed VRs. The loop (VR) that we designated Loop 6 was, in particular, characterised by a sustained run of low model consistency and high evolutionary rates (Fig 7).

We considered signatures of selection at codon sites within the amdoparvoviral species VP1 phylogeny, employing bootstrapped FEL and testing at the FVAV branch. As recombination analysis with GARD inferred up to 8 recombination breakpoints, across some of which the sister relationships of FVAV changed, the FEL analysis was partitioned at these breakpoints. Amongst 537 non-invariant codons, 5 were predicted to be under diversifying selection, 204 under purifying selection and 327 neutral (Fig 7). Signatures of selection were independently distributed amongst loop and non-loop structures in general, but sites under neutral or diversifying selection were significantly concentrated in Loop 6 vs the rest of the molecule (Fisher exact test, *P* = 0.02). Sites under diversifying selection were significantly clustered (Kolmogorov-Smirnov test, *P* < 0.001), with nearby pairs of sites occurring within the Loop 6 VR and in the N-terminal region, upstream of the polyglycine region.

Within the FVAV population in the Kielder Forest there were up to 6 amino acid differences in the VP1 protein (99.12% identity) between the available complete sequences, arising from 7 polymorphic sites (Fig 7). These polymorphic sites occurred at positions consistent with the interspecific pattern of variability described above, in all cases located within or flanking loop VRs, including one polymorphism in the Loop 6 VR. Analysis with GARD furthermore indicated the likelihood of up to 5 recombination breakpoints amongst the FVAV VP1 sequences.

### The FVAV non-structural protein (NS) is relatively fast-evolving and within-population mutation mirrors interspecific divergence

**Fig 9:**
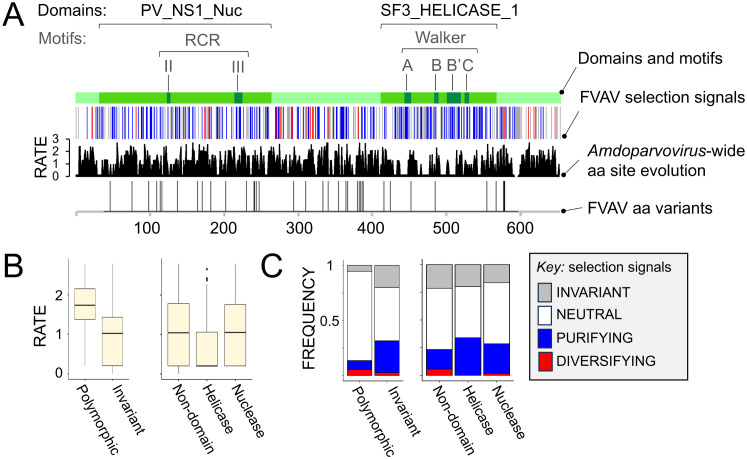
Structural variation in field vole amdoparvovirus (FVAV) NS1. **A**. From the top, tracks show the distribution of: protein domains and motifs; codon selection signatures; amino acid (aa) evolutionary rate derived from an *Amdoparvovirus*-wide alignment; the distribution of amino acid polymorphisms in the Kielder Forest FVAV population. Domain nomenclature follows PROSITE; PV_NS1_nuc, nuclease domain; SF3_HELICASE_1, helicase domain. For the evolutionary rate track the y-axis indicates the relative rate in substitutions per site. **B**. Box-and-whisker plots showing the distribution of evolutionary rate (derived from an *Amdoparvovirus*-wide alignment) across polymorphic and invariant sites in FVAV in the Kielder Forest (left-hand panel) and across FVAV nuclease and helicase domains and other sequence regions (right-hand panel). **C**. Stacked bar chart showing the relative frequency of different selection signatures (see key) across polymorphic and invariant sites in FVAV in the Kielder Forest (left-hand panel) and across the FVAV nuclease and helicase domains and other sequence regions (right-hand panel).

The sequence of the NS1, NS2 and NS3 proteins could be robustly inferred from the R2 mRNA- and R1’ mRNA-like contigs alone, on the basis of conserved splicing site residues, synteny and sequence homology with genomes and mRNA species reported in other amdoparvoviruses (Figs 1 and 9). As previously observed in other amdoparvoviruses, the NS proteins in FVAV are more diverged from those in close relatives than the VP proteins (for example, NS1 in FVAV shares c.70% amino acid identity with NS1 in RFFAV, whilst VP in FVAV shares c. 90% identity with VP in RFFAV). Nonetheless, NS1 has a highly conserved domain architecture, comprising an N-terminal nuclease domain with RCR II and RCR III motifs and a C-terminal helicase domain with ATP-binding Walker, A, B, B’ and C motifs (Fig 9).

As for VP1, analysis of synonymous and non-synonymous substitution in a genus-wide NS1 alignment (bootstrap FEL, testing at the FVAV branch) indicated a preponderance of purifying selection and neutral evolution at variable sites (19 diversifying, 162 purifying, 347 neutral) (Fig 9). This analysis was partitioned according to 16 recombination breakpoints inferred by GARD (across some of which the sister relationships of FVAV changed). Signatures of selection were distributed non-independently amongst the NS1 and VP1 proteins (Fisher exact test, *P* = 0.001), with NS1 having proportionately more sites under neutral and diversifying selection compared to VP1. Evolutionary rates (Kruskal-Wallis test, *P* = 6.5 × 10^-6^) and the distribution of selection signatures (Fisher exact test, *P* = 0.004) varied significantly across the NS1 domain and non-domain regions (Fig 9), with the helicase domain being more conserved and having relatively fewer sites under neutral or diversifying selection. Sites subject to diversifying selection were significantly clustered (Kolmogorov-Smirnov test, *P* < 0.001) and occurred outside the helicase domain (Fig 9).

Within the FVAV population in the Kielder Forest we found up to 29 amino acid differences (94.8% identity) in NS1 sequences between hosts, arising from 39 polymorphic sites (Fig 9). This was based on a 561 amino acid alignment for 5 sequences that excluded a short N-terminal segment of the common NS leader sequence and the C-terminal exon (in order to maximise the number of sequences analysed). The polymorphic sites mirrored the interspecific evolutionary pattern, being significantly associated with higher evolutionary rates in a genus-wide alignment (Fig 9) (Kruskal-Wallis test, *P* = 2.7 × 10^-8^). Polymorphic sites were non-randomly associated with the distribution of selection signatures (Fisher Exact test, *P* = 0.00020), polymorphisms being more likely at sites evolving neutrally or under diversifying selection. Furthermore, the polymorphic sites were significantly clustered (Kolmogorov-Smirnov test, *P* < 0.001), in particular being absent from a long region in the helicase domain. Analysis with GARD indicated the likelihood of up to 3 recombination breakpoints within the FVAV sequences alone.

### There are convergent host-specific signatures in the FVAV VP capsid molecular structure supporting that it is an arvicoline specialist

**Fig 10:**
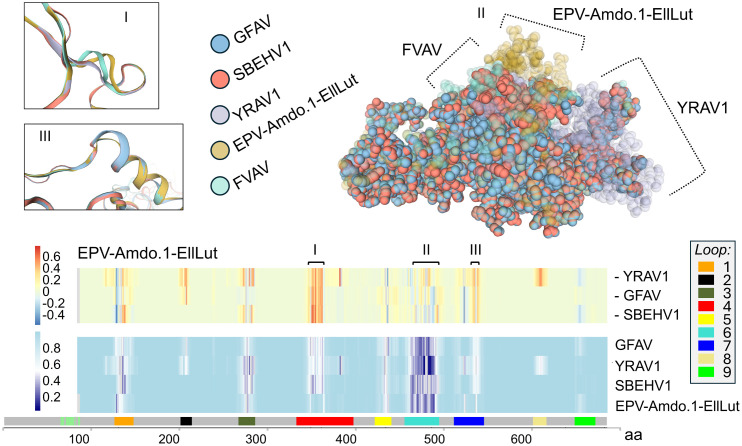
Host-specific features of the viral capsid. The structure of VP1 in field vole amdoparvovirus (FVAV) was more consistent with a phylogenetically divergent endogenized lineage also occurring in arvicoline rodent hosts, EPV-Amdo.1-EllLut, than it was with more closely related lineages in other hosts. VP1 molecular modelling results are presented for species clustering with, but falling outside, a well-supported VP clade of carnivoran-infecting amdoparvoviruses (see Fig 3) and also for endogenized amdoparvovirus (EPV-Amdo.1-EllLut) which clusters separately. Upper panels show superimposed species-specific VP1 models; lower panels show tracks representing regions and measurements along the linear amino acid sequence of VP1. Lower panels: tracks showing the position of FVAV motifs and loops (coloured as in Fig 8) (bottom), the aligned by-residue structural model consistency of each virus with FVAV (middle) (darker colours indicate low consistency, see colour scale on left) and the aligned difference in consistency with FVAV between EPV-Amdo.1-EllLut and each other virus in the FVAV cluster (redder colours indicate regions where FVAV is more consistent with EPV-Amdo.1-EllLut than with its cluster-mates; see colour scale on left) (top). The scale at the bottom indicates amino acid residue position along the linear sequence of VP1 in FVAV. Alignment gaps for non-FVAV lineages are shown in grey in the top two tracks. Upper panels: showing three-dimensional representations of modelled VP1 structures in regions (labelled, Roman numerals) where FVAV is more consistent with EPV-Amdo.1-EllLut than with cluster-mates. Superimposed molecular structures for different viral lineages are colour-coded (see key). Regions I (Loop 4) and III (Loop 7) show local differences in loop structures in a “cartoon” representation (representing the protein backbone). For region II (Loop 6), which encompasses an extended run of residues with indels, the entire superimposed VP1 structure is shown in a “spacefill” representation with FVAV, EPV-Amdo.1-EllLut and Yunnan rodent amdoparvovirus (YRAV1, a lineage occurring in murine rodents) atoms shown in transparency. (The spacefill representation shows atoms as spheres, with sizes proportional to their van der Waals radii.) Note that for region II, all of the rodent-infecting lineages have Loop 6 structures projecting in a similar plane compared to gray fox amdoparvovirus (GFAV; International Committee on Taxonomy of Viruses name: *Amdoparvovirus carnivoran2)* (canid hosts) and Sabeidhel virus (SBEHV1; *Amdoparvovirus chiropteran1*) (bat hosts). Furthermore, the Loop 6 structure in YRAV1 is larger and differently directed compared to those in FVAV and EPV-Amdo.1-EllLut, which are smaller and more similarly situated.

Given the dispersal of several non-monophyletic amdoparvoviral lineages amongst rodent hosts (Fig 3), we hypothesised that there could be rodent-specific structural adaptions of VP1 that might be independent of, and thus distinguishable from, phylogenetically conservative traits. We compared VP1 models between FVAV and other lineages clustering outside the main carnivore-associated VP cluster, including GFAV from canids, SBEHV1 from bats, and YRAV1 from murine rodents (Fig 3). We also compared the endogenized amdoparvovirus, EPV-Amdo.1-EllLut, which is more distantly related and falls outside the aforementioned cluster (Fig 3) but infects hosts in the same narrow rodent group as FVAV (cricetid rodents of the subfamily Arvicolinae). We found that, in fact, EPV-Amdo.1-EllLut, was the most structurally consistent with FVAV (mean = 0.93; GAMLSS, *P* << 0.001 in pairwise comparisons to other lineages), and YRAV1 (mean = 0.88) was the least consistent (Fig 10) despite the fact that FVAV never clusters together with EPV-Amdo.1-EllLut in analyses of primary sequences (see Fig 3). Thus, there appear to be phylogeny-independent arvicoline-specific features in the 3-dimensional molecular structure of VP1. Areas of shared higher consistency between FVAV and PVe-Amdo-EllLut.1 were widely distributed and included regions in Loops 4, 6 and 7, with the evolutionarily labile Loop 6 region in particular containing extended externally-facing features that were more similar in FVAV and EPV-Amdo.1-EllLut (see Fig 10) and particularly diverged in the murine-infecting YRAV1 due to an indel.

## Discussion

Although amdoparvoviruses have previously mainly been associated with carnivoran hosts, here we discovered an endemic, exogenous amdoparvovirus in wild arvicoline rodents (field voles) in Northumberland, Northern England. This finding is important because it extends our understanding of host utilization in amdoparvoviruses, which are a group associated with high transmissibility and high potential to cause disease [1]. Based on the full coding sequencing sequences recovered, the new virus, field vole amdoparvovirus (FVAV), is most similar to genomic fragments of RFFAV and EFAV-1 previously reported in the European red fox [92] and in wildcats [93] in the Basque country and Navarra in Northern Spain. The phylogenetic proximity of this relationship, within a generic tree mostly characterised by deep branches associated with distinct host groups, could be consistent with host transfers between rodent and carnivoran hosts, as has previously been suggested might occur through predator-prey contacts [94]. Nonetheless, the amino acid sequence divergence of the FVAV NS1 protein, at ≤ 70% identity in comparison to other lineages, lies far outside the *Amdoparvovirus* species demarcation criterion currently recommended by the ICTV [94] (85% identity). Thus, FVAV might be regarded a new species related to the RFFAV and EFAV-1 lineages found in Spanish red foxes and wildcats.

Within the wider *Amdoparvovirus* genus, Maximum Likelihood phylogenetic analyses of viral molecular sequences indicated that FVAV falls outside of a previously recognised, well-supported cluster of amdoparvoviruses that consistently infect carnivorans [9,93]. This carnivoran-associated cluster includes AMDV and related viruses occurring in procyonids, mephitids, viverrids, ailurids and canids across the Palearctic and Nearctic. Other amdoparvoviruses that, like FVAV, arise on deeper branches outside the carnivoran-associated cluster have more poorly resolved interrelationships and present a heterogenous host and geographical distribution. These viruses include ICTV-recognised species [89] in the North American gray fox (GFAV) [95] and West African bats (SBEHV1) [10] and metagenomic sequences detected in murine rodents (RtRn-ParV/GZ2026 and YRAV1) in China [14,15]. Further divergent lineages arise in Chinese bats (BtRl-PV/FJ2012) [12] and, as endogenized elements, in Transcaucasian mole voles (EPV-Amdo.1-EllLut and EPV-Amdo.2-EllLut) [13].

FVAV was detected at high prevalence in the field vole population in Northumberland via lung metatranscriptomics and real-time PCR targeting genomic DNA. Eighty nine percent (34/38) of voles were detectably infected in metatranscriptomic measurements and 100% via real-time PCR. Even if a cut-off based on a worst-case-scenario for contamination was applied to the metatransciptomic data, prevalence was still estimated at almost half of the population (47%, 18/38). Moreover, the real-time PCR viral DNA quantities were correlated with the metatranscriptomic reads, providing confidence that a genuine signature of infection in a substantial number of hosts was present. In fact, for both the metatranscriptomic and real-time PCR measurements, it is not possible to rule out that all hosts harboured some level of infection. Although viral mRNA was undetectable through metatranscriptomics in 11% (4/38) of hosts, the continuous left-hand frequency distribution of read counts approaching zero makes it difficult to determine (neglecting the possibility of contamination) whether this was due to sensitivity of measurement or genuine lack of infection.

We were able to assemble 6 complete or nearly complete sets of FVAV mRNA coding sequences from individual voles (where viral mRNA expression was high), all of which were unique and which clustered into 4 variants. The similarity of smaller coding fragments assembled from other hosts to these variants could further be determined, revealing that the variants co-circulated across the study period, with the most common variant being widespread throughout the spatiotemporal frame of the study. Short read mapping analysis in hosts with high viral expression indicated very low or absent coinfection with different variants and provided assurance that the variant sequences we assembled were not chimeric in nature (moreover, assemblers will tend to converge on a dominant consensus sequence). This was also supported by the virtual identity of the viral DNA genome sequenced in one host to the coding regions assembled from transcriptomic reads in the same host. Importantly, the distribution of the different viral variants broke across host mtDNA haplotypes and thus matrilineages. This lack of association between the viral variants and host matrilineages is consistent with a recent history of horizontal transmission, although a role for vertical transmission in contributing to the high observed prevalence cannot be ruled out.

The high infection prevalence observed for FVAV is consistent with chronic infection and a failure to fully clear infection, and/or high infection and reinfection rates. Moreover, the highly skewed viral transcript abundance distribution is suggestive of an initial acute phase of infection and/or a tendency to recrudesce. High expression of the virus was clearly positively associated with the upwards differential expression of a broad range of immune response pathways in host lung tissue. This is most consistent with an inflammatory cellular infiltration in more heavily infected lungs. Although such an inflammatory infiltration might be driven by FVAV infection and could be consistent with a level of disease caused by FVAV, nevertheless other scenarios are possible. For example, increased FVAV replication might, in fact, be a sequel to altered host immune activity resulting from a coinfection or another systemic perturbation. Genes associated with pneumonia and responses to oxidative stress were also upwardly expressed as viral expression increased. This is additionally consistent with covariance between FVAV replication and pulmonary inflammation, as the more prominently upregulated pneumonia-associated genes were pro-inflammatory in nature and oxidative stress is well known to be interconnected with inflammation [91]. In contrast, gene sets that might reveal deep-seated disease progression (e.g., cell cycle, apoptosis, metabolic process, pneumocyte development) were unperturbed as FVAV abundance increased, so that any severe disease caused by FVAV may be absent, or transient and difficult to observe. Furthermore, associations with FVAV infection extended beyond the lung, as we observed evidence of impaired splenic T-cell activation as pulmonary viral expression increased. As for the pulmonary transcriptomic responses above, we cannot be sure whether this association might be driven by FVAV, or alternatively whether FVAV replication may have instead been promoted by physiological responses that the host is making to other stimuli.

The genomic architecture and evolutionary pattern of FVAV appears to be mostly consistent with other members of the *Amdoparvovirus* genus [9,96]. As for other amdoparvoviruses, the capsid VP1 protein lacks a phospholipase A2 domain and contains a polyglycine region and several highly variable regions (VRs) located on loops that are directed towards the outside of the capsid polymer [9] where they are likely to interact with neutralising antibodies [31]. Overall, the VP1 sequence is dominated by purifying or neutral selection [96], with most variation in VRs generated by neutral processes. However, one of the most variable VRs (Loop 6), that also has particularly low structural similarity to other amdoparvoviruses in molecular models, contains a cluster of sites under diversifying selection in the FVAV lineage. This region, furthermore, maps onto previously described immunoreactive sites in AMDV [31,97,98], including a partly conserved N-terminal motif previously linked to antibody-dependent enhancement (ADE) [31]. A parsimonious explanation for the existence of the rapidly and predominantly neutrally-evolving VRs is that their high variability is permitted by a role in recognition by neutralising antibodies. As antibodies can adapt to recognise almost any molecular structure [99] and antibody binding may in some circumstances be beneficial for amdoparvovirus, via ADE [31,100], this may remove selective constraints and create a situation especially permissive for rapid neutral evolution. Another cluster of sites under diversifying selection in the FVAV lineage is found in the VP1 region upstream of the polyglycine sequence. This region, or the regions within the VRs that contain actively selected sites, could possibly be involved in host-specific adaptation and in coevolutionary interactions with the host [101,102].

As for other amdoparvoviruses, NS1 is considerably more variable than VP1 in FVAV and has more sites under diversifying selection but is still dominated by neutral and purifying processes [9,103]. The domain structure of NS1 is conserved [104], with a nuclease and a helicase domain containing similar functional motifs to those in other amdoparvoviruses. The helicase domain is relatively the most conserved, with higher evolutionary rates and clusters of sites under diversifying selection occurring in the nuclease domain and in regions outside the domains. As noted above, such areas, where there is evidence of adaptive selection, could be associated with coevolutionary interactions with host receptors or host defensive systems. For example, the nuclease domain of the mink enteritis parvovirus (MEV) NS1 has been reported to inhibit Type I IFN production [105].

Parvovirus mutation rates, although subject to uncertainty, are recognised to be relatively high [106,107]. Nonetheless, the molecular divergence observed [106,107] across the well-documented post-1971 canine parvovirus (*Protoparvovirus*) panzootic [8] or during the global spread of AMDV [108] would suggest that the most divergent currently detected FVAV lineages are at least several years old or more and are thus likely to have co-existed for some time. Co-circulation of multiple strains or variants, as observed in FVAV, often occurs in fast-evolving viruses [109] and may depend on immunological interactions with the host involving cross-immunity and evolutionary immune escape, including via recombination [110,111]. Given the observations above, of highly variable host-interacting structures, of high levels of recombination, and of FVAV-associated inflammation in the lung, it is certainly possible that FVAV could be involved in such immunological interactions.

The mutational pattern observed within the FVAV population mirrors that seen amongst species-level amdoparvoviral lineages. Thus, many more amino acid polymorphisms occurred in the NS than VP coding sequences, consistent with the greater divergence of NS amongst *Amdoparvovirus* species [9]. Within the NS region, for which the higher number of mutations allowed more detailed analysis, intrapopulation polymorphisms occurred at sites with higher *Amdoparvovirus*-wide evolutionary rate, were more likely to be sites previously under neutral or diversifying selection and were significantly clustered and absent from a long region in the conserved helicase domain. Even amongst the smaller number of mutations detected in FVAV VP1, these occurred within or flanking VRs. This consonance of intra- and inter-specific evolution suggests that processes shaping variation at the interspecies level may proceed directly from population processes taking place actively within established virus-host associations, rather than the two being categorically different processes.

Amdoparvoviruses have previously been associated with high levels of recombination [9,112]. This was supported for the VP and NS coding regions in FVAV, both within the recent radiation of FVAV and between FVAV and congeneric lineages. Thus, sister relationships changed along the VP and NS regions, both amongst conspecific FVAV lineages and amongst FVAV and heterospecific lineages. The existence of such recombination events is consistent with dynamic host usage over time at the interspecies level and with horizontal transmission at the intraspecies level, as both scenarios could result in viral lineages switching between, and coming into contact within, different hosts.

Molecular modelling of VP1 in different *Amdoparvovirus* species suggested that the three-dimensional structure of FVAV VP1 was most consistent with that of an endogenised lineage (EPV-Amdo.1-EllLut) in Transcaucasian mole voles [13]. Mole voles, like the field vole hosts of FVAV, are cricetid rodents from the subfamily Arvicolinae. The three-dimensional similarity between FVAV and EPV-Amdo.1-EllLut VP1 occurs despite the fact that, at the primary sequence level, FVAV is dissimilar to EPV-Amdo.1-EllLut and always clusters more closely to other lineages in a range of non-arvicoline hosts. Across several three-dimensional VP1 features, including in the Loop 6 region, FVAV is consistently less similar to these lineages and more similar to EPV-Amdo.1-EllLut, despite the relationships based on primary sequences. This may be an instance of evolutionary convergence, as previously described in the capsid of other *Parvoviridae* [113] whereby, in the present case, divergent lineages infecting arvicolines may have independently evolved similar capsid structures. This would tend to support that FVAV is an arvicoline specialist.

In summary, we have demonstrated the presence of an endemic, high-prevalence amdoparvovirus in wild arvicoline rodents (field voles). The new virus (FVAV) likely has at least some component of horizontal transmission and is associated with some level of pulmonary and systemic disease in its hosts. These observations extend the previously known flexibility of host usage of the *Amdoparvovirus* genus and are consistent with its known disease-causing tendencies. As FVAV is most closely related to amdoparvoviruses in red foxes and wildcats [92,93], which are natural predators of the field vole [114–116], more work is required to establish whether FVAV can additionally exploit the carnivoran predators of field voles. Nonetheless, FVAV demonstrated convergences in capsid structure with amdoparvovirus endogenized in other arvicolines [13] (mole voles), which supports that it is an arvicoline specialist. Importantly, the methods we have employed here, revolving around the *de novo* sequence assembly of viral RNA products in rRNA-depleted total RNA from tropic tissues in individual hosts, appear a sensitive and underappreciated way to detect and characterise both RNA and DNA viruses [35]. The immediate discovery of novel pulmonary viruses by applying these methods [35] suggests that the pulmonary virome of common wild mammals is likely to be greatly underestimated and under-surveyed. As respiratory system viruses represent a particular risk for consequential transboundary emergence [117], improved surveys of the pulmonary virome in common wild mammals (and other warm-blooded animals), conducted with sensitive and biologically informative methods such as those applied here, are urgently required. Such survey data would help evaluate transboundary risks to animal and human health and would provide a baseline against which future changes due to environmental fluctuation or anthropogenic introductions could be evaluated.

## Supporting information

S1 Table Gene sets for GSEA analysis. (XLSX)

S2 Table Random Forest predictors and variable importance. (XLSX)

S3 Table Primers for amplification of viral genomic DNA. (PDF)

S4 Table Table of FVAV genomic DNA, mRNA and amino acid sequences analysed. (PDF)

S5 Table Breakdown of host study sample characteristics. (PDF)

S6 Table Pulmonary RNAseq differentially expressed genes. (XLSX)

S7 Table GSEA analysis results. (XLSX)
