# Supplementary material for: Exogenous amdoparvoviruses (Parvoviridae) in arvicoline voles: the molecular evolution and ecology of a novel host-viral association
Source: PLoS Pathog. 2026 Jan 22;22(1):e1013896. doi: 10.1371/journal.ppat.1013896 (PMC12863673; doi:10.1371/journal.ppat.1013896)
Supplement: S3 Table — (PDF) [file ppat.1013896.s003.pdf]

SUPPLEMENTARY TABLE S3

Table S3. Primers for amplification of viral genomic DNA

Primer identifier	Sequence
LSTART	ATGGCACAGGCTCAGC
L2	GTTTGAACAGCTGAAGAGGG
L3	AGAAGAGACTGAGAGACCTGT
L4	GGGATGCAACCAAAGTAACCC
L5	TGGAAATGACATACTGAAGCCA
L6	GCCAGCAACAAAACCACAAA
L7	GGCTGCATTTGGTTCTTTGG
L8	CCCATCAACCAAAGTAACACCA
L9	ACTGGACCAAACTTGACTGTG
L10	GCTGCTGAGGTTGCTGAAAA
L11	ATACACTCCAGCAGCTCCAC
L12	AGGACTGCAGGGAAGTTACT
LEND	GGGACAAGAAGCCAGACATG
R3	TTGATGTCTTTTGGGTCGCC
R2_4	CCACCATTTCCAGCAGCAAA
R5	CCCATTTGCTTCAAGGCTCA
R6	ATCAACCTTTACGCTGGCAC
R7	TTTGTCCCAATCACTTAGCCA
R8	AGTTTGTTGTGCTCGCAGTT
R9	GGTGATATTTCCCTGCCTGC
R10	GGTTGGTTTGGTTGCTCTCC
R11	TGGTTGTGTCACTCCATTGC
R12	TGTCTGGATCCTCATCATGGT
REND	GTAGATGTATTTAACAGTACTTCTTCC
FAREND	TTTCCCTACACGACGCTCTT
