# Supplementary material for: Exogenous amdoparvoviruses (Parvoviridae) in arvicoline voles: the molecular evolution and ecology of a novel host-viral association
Source: PLoS Pathog. 2026 Jan 22;22(1):e1013896. doi: 10.1371/journal.ppat.1013896 (PMC12863673; doi:10.1371/journal.ppat.1013896)
Supplement: S4 Table — (PDF) [file ppat.1013896.s004.pdf]

SUPPLEMENTARY TABLE S4

Table S4. Table of field vole amdoparvovirus (FVAV) genomic DNA, mRNA and amino acid sequences analysed.

Host ID	Variant	Name	Molecule type	Completeness (coding sequences)	Sequence length*	GenBank accession
V362	2	R1’	pre-mRNA	Partial	1822	PX514923
V415	3	R1’	pre-mRNA	Partial	1821	PX514924
V427	4	R1’	pre-mRNA	Partial	1822	PX514925
V512	4	R1’	pre-mRNA	Partial	1821	PX514926
V531	4	R1’	pre-mRNA	Partial	1821	PX514927
V280	1	R2	mRNA	Partial	2025	PX514917
V362	2	R2	mRNA	Near complete	2483	PX514918
V415	3	R2	mRNA	Complete	2491	PX514919
V427	4	R2	mRNA	Near complete	2608	PX514920
V512	4	R2	mRNA	Near complete	2479	PX514921
V531	4	R2	mRNA	Near complete	2494	PX514922
V280	1	VP1/VP2	Protein	Partial	648	
V362	2	VP1/VP2	Protein	Complete	685	
V415	3	VP1/VP2	Protein	Complete	685	YBR23470
V427	4	VP1/VP2	Protein	Complete	685	
V512	4	VP1/VP2	Protein	Complete	685	
V531	4	VP1/VP2	Protein	Complete	685	
V362	2	NS1	Protein	Partial	561	
V415	3	NS1	Protein	Complete	654	YBR23467
V427	4	NS1	Protein	Partial	561	
V512	4	NS1	Protein	Partial	561	
V531	4	NS1	Protein	Partial	561	
V415	3	NS2	Protein	Complete	114	YBR23468
V415	3	NS3	Protein	Complete	71	YBR23469
V415	3	Genome	DNA	Near complete	4157	PX491703
