# Supplementary material for: Exogenous amdoparvoviruses (Parvoviridae) in arvicoline voles: the molecular evolution and ecology of a novel host-viral association
Source: PLoS Pathog. 2026 Jan 22;22(1):e1013896. doi: 10.1371/journal.ppat.1013896 (PMC12863673; doi:10.1371/journal.ppat.1013896)
Supplement: S5 Table — (PDF) [file ppat.1013896.s005.pdf]

SUPPLEMENTARY TABLE S5

Table S5. Breakdown of sample characteristics for study animals. Sample means, standard deviations (in parentheses) and ranges are given for vole weight (pre-dissection) and body length (snout to vent). Also given are the percentage prevalence (P) of infection with field vole amdoparvovirus (FVAV) detectable via RNA sequencing of lung samples and the abundance (A) of FVAV reads (mean, standard deviation in parentheses and range) normalised to the mean library size of 31 million reads. The dataset is broken down into compartments representing life history stages, season and site (BLB/CHE/GRD/HAM/SCP).

Data compartment	N	Weight (g)	Length (mm)	P	A
Juvenile female	2	18 (1) 17-19	85 (2) 83-86	50	3 (4) 0-5
Mature female	9	27 (6) 20-38	103 (8) 91-116	100	77 (205) 1-624
Mature male	27	37 (6) 26-49	112 (18) 99-122	89	406 (979) 0-3905
Summer-Autumn 2016	9	24 (4) 18-29	98 (10) 83-117	89	54 (132) 0-406
Spring 2017*	16	39 (7) 23-49	113 (5) 104-122	88	513 (1006) 3-3056
Summer 2017	13	34 (6) 21-43	111 (9) 91-121	92	300 (935) 0-3905
BLB	20	37 (8) 21-49	111 (8) 91-122	95	372 (948) 0-3905
CHE	5	29 (8) 18-38	105 (14) 83-121	80	11 (9) 0-22
GRD	2	32 (4) 28-35	106 (6) 102-111	50	0 (1) 0-1
HAM	7	36 (6) 26-42	113 (5) 107-121	100	507 (1135) 8-3056
SCP	4	22 (3) 19-26	96 (8) 86-105	75	159 (310) 0-625
All	38	34 (8) 18-49	109 (10) 83-122	89	307 (841) 0-3905

*Seasons were demarcated by the astronomical calendar but one individual from late winter 2016-2017 (14th March 2017) is included in the spring 2017 sample for simplicity.
